# Crystal structure of (*E*)-4,4′-(but-2-ene-1,4-di­yl)bis­(2-meth­oxy­phenol)

**DOI:** 10.1107/S2056989015011585

**Published:** 2015-06-20

**Authors:** Kyle S. Knight, Patrick J. Carey

**Affiliations:** a Department of Chemistry, The University of Tennessee at Chattanooga, Chattanooga, TN 37403, USA

**Keywords:** crystal structure, metathesis, dimerization of eugenol, hydrogen bonding

## Abstract

The title compound, C_18_H_20_O_4_, was synthesized *via* the ruthenium-catalyzed alkene methathesis dimerization of eugenol. The whole mol­ecule is generated by inversion symmetry; the center of inversion being located at the mid-point of the *trans* C=C bond. The phenol ring is inclined to the mean plane of the central C—C=C—C unit (r.m.s. deviation = 0.014 Å) by 68.83 (16)°. In the crystal, mol­ecules are linked *via* O—H⋯O hydrogen bonds, involving the hy­droxy and meth­oxy groups, forming undulating sheets parallel to (010).

## Related literature   

For a general review of alkene metathesis catalyzed by ruthenium carbenes, see: Grubbs (2004 ▸). For the second generation Grubbs ruthenium carbene catalyst, see: Scholl *et al.* (1999 ▸). For the synthesis of the title compound, see: Taber & Frankowski (2006 ▸).
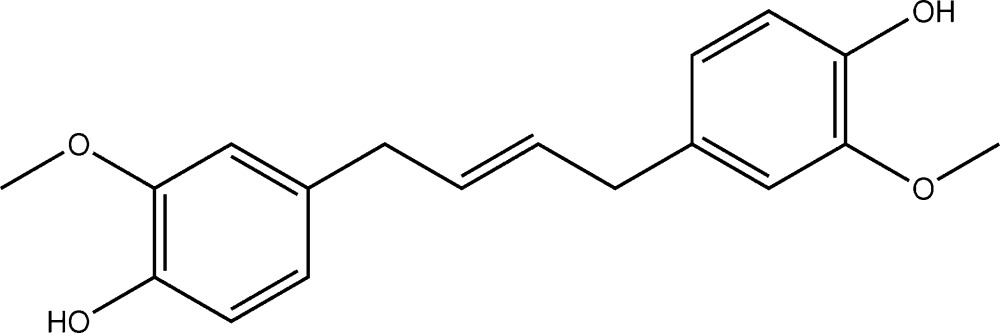


## Experimental   

### Crystal data  

C_18_H_20_O_4_
*M* *_r_* = 300.34
Orthorhombic, 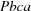

*a* = 4.8846 (2) Å
*b* = 10.7002 (4) Å
*c* = 29.5666 (11) Å
*V* = 1545.33 (10) Å^3^
*Z* = 4
Mo *K*α radiation
μ = 0.09 mm^−1^
*T* = 198 K
0.6 × 0.55 × 0.2 mm

### Data collection  

Bruker APEXII CCD diffractometer
Absorption correction: multi-scan (*SADABS*; Bruker, 2009 ▸) *T*_min_ = 0.927, *T*_max_ = 1.000
25610 measured reflections
1352 independent reflections
1199 reflections with *I* > 2σ(*I*)
*R*_int_ = 0.036

### Refinement  

*R*[*F* ^2^ > 2σ(*F* ^2^)] = 0.033
*wR*(*F* ^2^) = 0.096
*S* = 1.05
1352 reflections
105 parameters
H-atom parameters constrained
Δρ_max_ = 0.16 e Å^−3^
Δρ_min_ = −0.13 e Å^−3^

### 

Data collection: *APEX2* (Bruker, 2009 ▸); cell refinement: *SAINT* (Bruker, 2009 ▸); data reduction: *SAINT*; program(s) used to solve structure: *SHELXS2014* (Sheldrick, 2008 ▸); program(s) used to refine structure: *SHELXL2014* (Sheldrick, 2015 ▸); molecular graphics: *OLEX2* (Dolomanov *et al.*, 2009 ▸) and *Mercury* (Macrae *et al.*, 2008 ▸); software used to prepare material for publication: *OLEX2*.

## Supplementary Material

Crystal structure: contains datablock(s) Global, I. DOI: 10.1107/S2056989015011585/su5153sup1.cif

Structure factors: contains datablock(s) I. DOI: 10.1107/S2056989015011585/su5153Isup2.hkl

Supporting information file. DOI: 10.1107/S2056989015011585/su5153Isup3.cdx

**Fig. 1.**
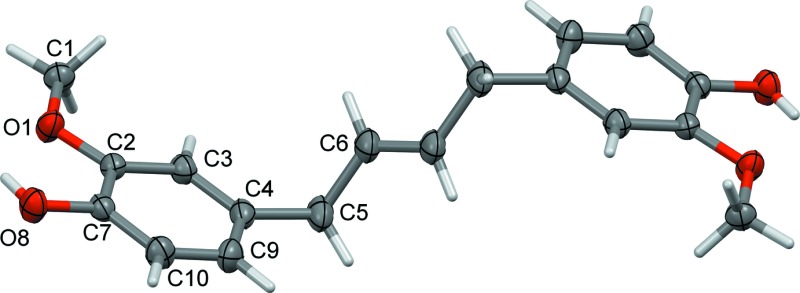
A view of the molecular structure of the title compound, with atom labelling. Displacement ellipsoids are drawn at the 50% probability level. Unlabelled atoms are related to the labelled atoms by inversion symmetry (symmetry code: -x + 1, -y + 2, -z).

**Fig. 2.**
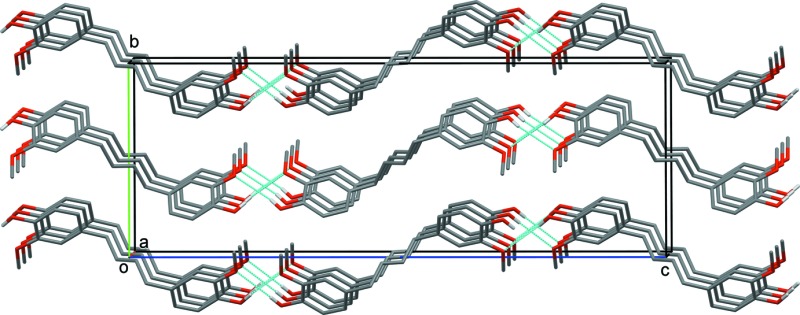
A view along the a axis of the crystal packing of the title compound, with hydrogen bonds shown as dashed lines (see Table 1 for details). C-bound H atoms have been omitted for clarity.

Supporting information file. DOI: 10.1107/S2056989015011585/su5153Isup4.cml

x y z . DOI: 10.1107/S2056989015011585/su5153fig1.tif
A view of the mol­ecular structure of the title compound, with atom labelling. Displacement ellipsoids are drawn at the 50% probability level. Unlabelled atoms are related to the labelled atoms by inversion symmetry (symmetry code: −*x* + 1, −*y* + 2, −*z*).

a . DOI: 10.1107/S2056989015011585/su5153fig2.tif
A view along the *a* axis of the crystal packing of the title compound, with hydrogen bonds shown as dashed lines (see Table 1 for details). C-bound H atoms have been omitted for clarity.

CCDC reference: 1406832

Additional supporting information:  crystallographic information; 3D view; checkCIF report

## Figures and Tables

**Table 1:** Hydrogen-bond geometry (, )

*D*H*A*	*D*H	H*A*	*D* *A*	*D*H*A*
O8H8O1^i^	0.78(2)	2.57(2)	3.1784(13)	136(1)

Symmetry code: (i) 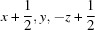.

## supplementary crystallographic information

### S1. Synthesis and crystallization

The title compound was prepared from eugenol by alkene metathesis dimerization using the second generation Grubbs ruthenium carbene catalyst (Scholl *et al.*, 1999) as described previously (Taber & Frankowski, 2006).

### S2. Refinement

Crystal data, data collection and structure refinement details are summarized in Table 2. The hydroxyl H atom was located in a difference Fourier map and refined with U_iso_(H) = 1.2U_eq_(O). The C-bound H atoms were positioned geometrically and constrained to ride on their parent atoms: C—H = 0.95 - 1.0 Å with U_iso_(H) = 1.5U_eq_(C) for methyl H atoms and 1.2U_eq_ (C) for other H atoms.

### Crystal data

**Table:**

C_18_H_20_O_4_	*D*_x_ = 1.291 Mg m^−^^3^
*M**_r_* = 300.34	Mo *K*α radiation, λ = 0.71073 Å
Orthorhombic, *P**b**c**a*	Cell parameters from 9944 reflections
*a* = 4.8846 (2) Å	θ = 2.8–24.8°
*b* = 10.7002 (4) Å	µ = 0.09 mm^−^^1^
*c* = 29.5666 (11) Å	*T* = 198 K
*V* = 1545.33 (10) Å^3^	Plate, colorless
*Z* = 4	0.6 × 0.55 × 0.2 mm
*F*(000) = 640	

### Data collection

**Table:**

Bruker APEXII CCD diffractometer	1199 reflections with *I* > 2σ(*I*)
Graphite monochromator	*R*_int_ = 0.036
φ and ω scans	θ_max_ = 25.0°, θ_min_ = 2.8°
Absorption correction: multi-scan (*SADABS*; Bruker, 2009)	*h* = −5→5
*T*_min_ = 0.927, *T*_max_ = 1.000	*k* = −12→12
25610 measured reflections	*l* = −35→35
1352 independent reflections	

### Refinement

**Table:**

Refinement on *F*^2^	Secondary atom site location: difference Fourier map
Least-squares matrix: full	Hydrogen site location: mixed
*R*[*F*^2^ > 2σ(*F*^2^)] = 0.033	H-atom parameters constrained
*wR*(*F*^2^) = 0.096	*w* = 1/[σ^2^(*F*_o_^2^) + (0.0476*P*)^2^ + 0.352*P*] where *P* = (*F*_o_^2^ + 2*F*_c_^2^)/3
*S* = 1.05	(Δ/σ)_max_ < 0.001
1352 reflections	Δρ_max_ = 0.16 e Å^−^^3^
105 parameters	Δρ_min_ = −0.13 e Å^−^^3^
0 restraints	Extinction correction: *SHELXL2014* (Sheldrick, 2015), Fc^*^=kFc[1+0.001xFc^2^λ^3^/sin(2θ)]^-1/4^
Primary atom site location: structure-invariant direct methods	Extinction coefficient: 0.009 (3)

### Special details

**Table:**

Geometry. All e.s.d.'s (except the e.s.d. in the dihedral angle between two l.s. planes) are estimated using the full covariance matrix. The cell e.s.d.'s are taken into account individually in the estimation of e.s.d.'s in distances, angles and torsion angles; correlations between e.s.d.'s in cell parameters are only used when they are defined by crystal symmetry. An approximate (isotropic) treatment of cell e.s.d.'s is used for estimating e.s.d.'s involving l.s. planes.

### Fractional atomic coordinates and isotropic or equivalent isotropic displacement parameters (Å^2^)

**Table:**

	*x*	*y*	*z*	*U*_iso_*/*U*_eq_	
O1	0.3753 (2)	0.94521 (9)	0.20535 (3)	0.0531 (3)	
C1	0.1882 (3)	1.04703 (13)	0.20379 (5)	0.0505 (4)	
H1A	0.2599	1.1120	0.1837	0.076*	
H1B	0.1646	1.0815	0.2343	0.076*	
H1C	0.0111	1.0178	0.1924	0.076*	
C2	0.4437 (3)	0.88991 (11)	0.16499 (4)	0.0395 (3)	
C3	0.3285 (3)	0.91684 (12)	0.12337 (4)	0.0454 (3)	
H3	0.1922	0.9798	0.1211	0.054*	
C4	0.4108 (3)	0.85237 (13)	0.08473 (4)	0.0475 (4)	
C5	0.2924 (3)	0.88644 (16)	0.03888 (4)	0.0605 (4)	
H5A	0.3136	0.8146	0.0181	0.073*	
H5B	0.0941	0.9035	0.0422	0.073*	
C6	0.4294 (3)	0.99880 (15)	0.01871 (4)	0.0554 (4)	
H6	0.4116	1.0754	0.0347	0.066*	
C7	0.6459 (3)	0.79875 (11)	0.16851 (4)	0.0419 (3)	
O8	0.7636 (2)	0.77137 (10)	0.20946 (3)	0.0556 (3)	
H8	0.699 (3)	0.8143 (16)	0.2278 (6)	0.067*	
C9	0.6067 (3)	0.76024 (14)	0.08901 (4)	0.0552 (4)	
H9	0.6619	0.7143	0.0631	0.066*	
C10	0.7244 (3)	0.73365 (14)	0.13057 (5)	0.0536 (4)	
H10	0.8598	0.6702	0.1329	0.064*	

### Atomic displacement parameters (Å^2^)

**Table:**

	*U*^11^	*U*^22^	*U*^33^	*U*^12^	*U*^13^	*U*^23^
O1	0.0688 (7)	0.0527 (6)	0.0378 (5)	0.0162 (5)	−0.0078 (4)	−0.0042 (4)
C1	0.0538 (8)	0.0470 (8)	0.0505 (8)	0.0073 (6)	0.0032 (6)	−0.0004 (6)
C2	0.0455 (7)	0.0381 (6)	0.0349 (6)	−0.0030 (5)	−0.0011 (5)	0.0016 (5)
C3	0.0491 (8)	0.0459 (7)	0.0413 (7)	0.0003 (6)	−0.0055 (5)	0.0057 (6)
C4	0.0534 (8)	0.0541 (8)	0.0349 (7)	−0.0139 (7)	−0.0006 (5)	0.0057 (5)
C5	0.0676 (10)	0.0772 (10)	0.0368 (7)	−0.0170 (8)	−0.0082 (6)	0.0073 (6)
C6	0.0660 (10)	0.0653 (9)	0.0348 (6)	−0.0031 (8)	−0.0081 (6)	0.0054 (6)
C7	0.0456 (7)	0.0415 (7)	0.0386 (6)	−0.0018 (5)	−0.0004 (5)	0.0071 (5)
O8	0.0644 (7)	0.0597 (6)	0.0426 (6)	0.0163 (5)	−0.0077 (5)	0.0057 (4)
C9	0.0643 (9)	0.0621 (9)	0.0393 (7)	−0.0016 (7)	0.0112 (6)	−0.0037 (6)
C10	0.0564 (8)	0.0544 (8)	0.0500 (8)	0.0109 (7)	0.0084 (6)	0.0038 (6)

### Geometric parameters (Å, º)

**Table:**

O1—C1	1.4228 (16)	C5—H5A	0.9900
O1—C2	1.3732 (15)	C5—H5B	0.9900
C1—H1A	0.9800	C5—C6	1.500 (2)
C1—H1B	0.9800	C6—C6^i^	1.304 (3)
C1—H1C	0.9800	C6—H6	0.9500
C2—C3	1.3834 (17)	C7—O8	1.3720 (15)
C2—C7	1.3921 (18)	C7—C10	1.3751 (19)
C3—H3	0.9500	O8—H8	0.777 (17)
C3—C4	1.3938 (18)	C9—H9	0.9500
C4—C5	1.5183 (17)	C9—C10	1.386 (2)
C4—C9	1.380 (2)	C10—H10	0.9500
			
C2—O1—C1	117.26 (10)	H5A—C5—H5B	107.9
O1—C1—H1A	109.5	C6—C5—C4	112.18 (12)
O1—C1—H1B	109.5	C6—C5—H5A	109.2
O1—C1—H1C	109.5	C6—C5—H5B	109.2
H1A—C1—H1B	109.5	C5—C6—H6	116.9
H1A—C1—H1C	109.5	C6^i^—C6—C5	126.11 (19)
H1B—C1—H1C	109.5	C6^i^—C6—H6	116.9
O1—C2—C3	125.76 (12)	O8—C7—C2	120.87 (11)
O1—C2—C7	114.17 (10)	O8—C7—C10	119.65 (12)
C3—C2—C7	120.07 (11)	C10—C7—C2	119.46 (11)
C2—C3—H3	119.7	C7—O8—H8	108.7 (13)
C2—C3—C4	120.59 (13)	C4—C9—H9	119.5
C4—C3—H3	119.7	C4—C9—C10	121.05 (12)
C3—C4—C5	120.21 (13)	C10—C9—H9	119.5
C9—C4—C3	118.58 (12)	C7—C10—C9	120.23 (13)
C9—C4—C5	121.19 (13)	C7—C10—H10	119.9
C4—C5—H5A	109.2	C9—C10—H10	119.9
C4—C5—H5B	109.2		
			
O1—C2—C3—C4	−178.58 (12)	C3—C2—C7—C10	−1.74 (19)
O1—C2—C7—O8	−1.02 (18)	C3—C4—C5—C6	80.50 (17)
O1—C2—C7—C10	177.67 (12)	C3—C4—C9—C10	−1.4 (2)
C1—O1—C2—C3	−6.20 (19)	C4—C5—C6—C6^i^	116.9 (2)
C1—O1—C2—C7	174.44 (11)	C4—C9—C10—C7	0.4 (2)
C2—C3—C4—C5	−177.32 (12)	C5—C4—C9—C10	176.73 (13)
C2—C3—C4—C9	0.8 (2)	C7—C2—C3—C4	0.75 (19)
C2—C7—C10—C9	1.2 (2)	O8—C7—C10—C9	179.89 (13)
C3—C2—C7—O8	179.57 (12)	C9—C4—C5—C6	−97.55 (17)

Symmetry code: (i) −*x*+1, −*y*+2, −*z*.

### Hydrogen-bond geometry (Å, º)

**Table:**

*D*—H···*A*	*D*—H	H···*A*	*D*···*A*	*D*—H···*A*
O8—H8···O1^ii^	0.78 (2)	2.57 (2)	3.1784 (13)	136 (1)

Symmetry code: (ii) *x*+1/2, *y*, −*z*+1/2.
