# Supplementary figures and images for: Crystal structure of (E)-4,4′-(but-2-ene-1,4-di­yl)bis­(2-meth­oxy­phenol)
Source: Acta Crystallogr E Crystallogr Commun. 2015 Jun 20;71(Pt 7):o500. doi: 10.1107/S2056989015011585 (PMC4518983; doi:10.1107/S2056989015011585)

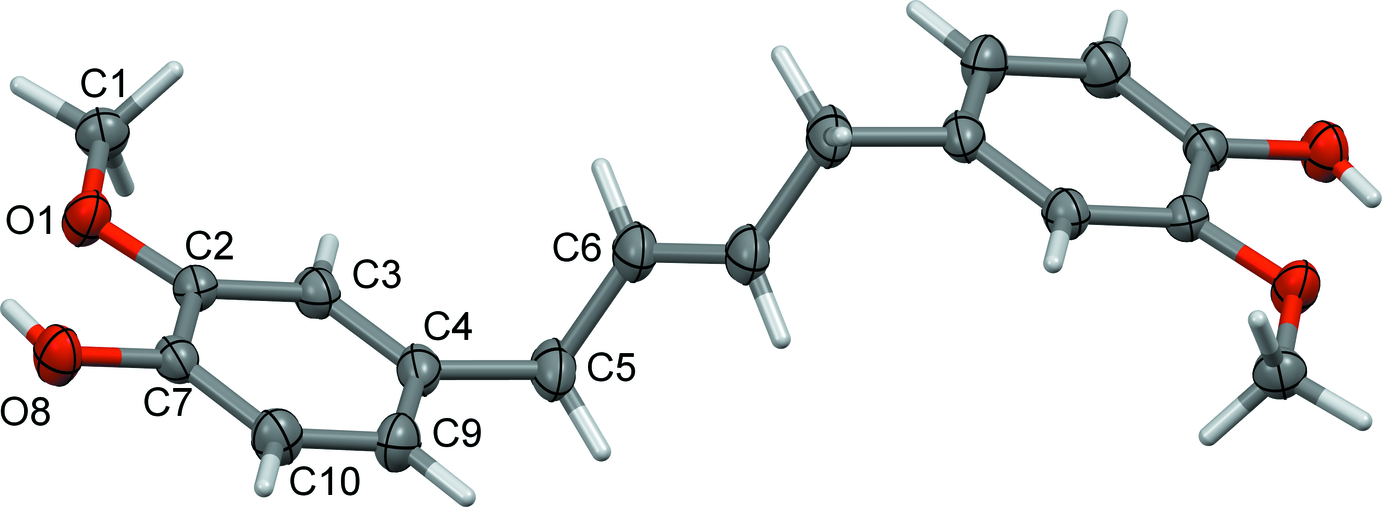

Supplement: Supplementary file 5 [file e-71-0o500-fig1.tif]

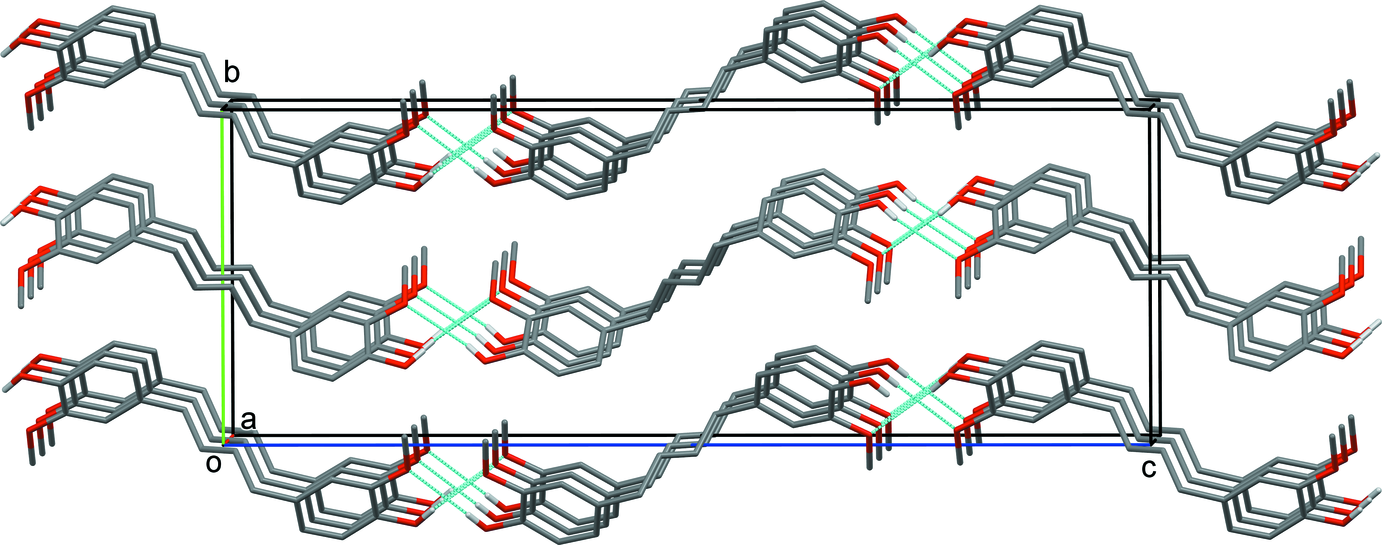

Supplement: Supplementary file 6 [file e-71-0o500-fig2.tif]
